# Bayesian analysis of heterogeneous treatment effects for patient-centered outcomes research

**DOI:** 10.1007/s10742-016-0159-3

**Published:** 2016-09-20

**Authors:** Nicholas C. Henderson, Thomas A. Louis, Chenguang Wang, Ravi Varadhan

**Affiliations:** 1 Sidney Kimmel Comprehensive Cancer Center, Johns Hopkins University, Baltimore, MD USA; 2 Department of Biostatistics, Johns Hopkins Bloomberg School of Public Health, Baltimore, MD USA

**Keywords:** Bayesian subgroup analysis, Heterogeneity of treatment effect, Hierarchical modeling, Personalized medicine, Precision medicine, Treatment–covariate interaction

## Abstract

Evaluation of heterogeneity of treatment effect (HTE) is an essential aspect of personalized medicine and patient-centered outcomes research. Our goal in this article is to promote the use of Bayesian methods for subgroup analysis and to lower the barriers to their implementation by describing the ways in which the companion software **beanz** can facilitate these types of analyses. To advance this goal, we describe several key Bayesian models for investigating HTE and outline the ways in which they are well-suited to address many of the commonly cited challenges in the study of HTE. Topics highlighted include shrinkage estimation, model choice, sensitivity analysis, and posterior predictive checking. A case study is presented in which we demonstrate the use of the methods discussed.

## Introduction

The conventional focus of clinical trials has been on assessing the average effect of a treatment in a target population. However, examining only the average treatment effect in the presence of patient heterogeneity may mask important differences in treatment efficacy or in treatment safety across subsets of patients. Heterogeneity of treatment effect (HTE) refers to differences in treatment effectiveness attributable to observable patient attributes such as demographic characteristics, genetic characteristics, and other baseline risk factors. For many diseases, understanding the extent and nature of treatment effect heterogeneity is key to the development of improved treatment strategies more tailored to individual patient needs.

Heterogeneous treatment effects are typically explored by examining patient outcomes in mutually exclusive subgroups defined by observable patient characteristics. In cases where a beneficial overall treatment effect has been found, such subgroup analyses are performed to examine the consistency of the claimed treatment effect across major patient sub-populations. Existence of subgroups that appear to respond differently to treatment can affect inclusion criteria in later clinical trials or in labeling decisions for approved drugs (Alosh et al. 2015). Though subgroup analyses are often recommended and routinely performed, there are a number of concerns which lead many to interpret the results of subgroup analyses with caution. As highlighted by many authors (Yusuf et al. 1991; Wang et al. 2007; Berger et al. 2014), these include problems related to multiplicity, post-hoc analyses performed after seeing the data (“data-dredging”), and small within-subgroup sample sizes. Proper adjustments for multiplicity in subgroup analysis can result in substantial loss of power to detect differences across subgroups, and small subgroup-specific sample sizes lead to highly variable estimates which frequently makes it challenging to assess the source and magnitude of HTE. In addition to concerns about low power and post-hoc analyses, conventional approaches to subgroup analysis have difficulty in characterizing variation in treatment effect after a treatment interaction has been determined to be present.

A Bayesian approach to HTE can address many of the common concerns with subgroup analysis while also providing more informative characterizations of HTE. Among the more compelling reasons to employ Bayesian methods for subgroup analysis is their excellent estimation performance in multi-parameter settings. A key feature of most Bayesian approaches to subgroup analysis is the inclusion of all subgroup-level treatment effects in one joint model. Incorporating all treatment effects in one common probability model, allows inferences in each subgroup to be driven by all the data rather than only the data in that particular subgroup. This has the consequence of stabilizing highly variable subgroup effect estimates by allowing these highly variable cases to “borrow information” from the data in other subgroups. Moreover, the borrowing of information across subgroups increases the precision of the individual estimates. The utilization of all the data alleviates to a large extent the common problem in subgroup analysis of small within-subgroup sample sizes and highly variable estimates. Indeed, these advantages of using Bayes estimates in multi-parameter settings have been widely acknowledged (Efron and Morris 1973; James and Stein 1961) and represents one of the main areas in which Bayesian methods can offer an improvement over other methods.

In addition to improved estimation and precision, the Bayesian framework can arguably provide answers to questions more in line with the goals of personalized/patient-centered medicine. Bayesian approaches naturally lend themselves to characterizations of HTE because such heterogeneity may be directly expressed through the distribution of the subgroup treatment effect parameters. While other approaches emphasize hypothesis testing and detection of HTE, the Bayesian models discussed here have the built-in assumption that HTE is present, and the statistical challenge is to estimate and characterize this heterogeneity in treatment effect. The implications of the estimated variation in treatment effect can then be evaluated taking into account posterior uncertainty, the prior information used, and the context of the problem. The Bayesian framework is also useful in its ability to automatically give direct probability statements to a wide range of questions of clinical interest. For example, from a personalized medicine perspective, a natural question to ask is: what is the probability that individuals from a particular subgroup will benefit from this treatment? A direct answer to such a question can be obtained from the full posterior distribution without the need to refer to repeated sampling characteristics as in frequentist inference. Addressing complex clinical questions such as these and providing associated uncertainty measures is often challenging when operating outside the Bayesian framework.

Our goal in this article is to promote the use of Bayesian methods for subgroup analysis and to lower the barriers to their implementation by describing the ways in which the companion software **beanz** can facilitate these types of analyses. To this end, we provide in this paper an overview of the models implemented by the **beanz** software tool (Wang et al. 2016), describe their merits, and outline other important factors to consider when using Bayesian methods for subgroup analysis. The web-based software tool **beanz** can be accessed from https://www.research-it.onc.jhmi.edu/dbb/custom/A6/, and the R package version of this software entitled **beanz** is available from the Comprehensive R Archive Network (http://cran.r-project.org). Information regarding the usage and capabilities of **beanz** is provided in greater detail in Wang et al. (2016), and a **beanz** software manual is also available from the **beanz** website. This article is organized as follows. In Sect. 2, we begin by introducing our motivating example—the SOLVD trial—and describe the key patient subgroups to be investigated in our data analysis. In Sect. 3, we then review several of the more conventional, frequentist approaches to subgroup analysis and examine their application to the SOLVD trial. In Sect. 4, we introduce several Bayesian models suggested in Jones et al. (2011) that can be used in subgroup analysis and describe several of their prominent features. Throughout this section, the Bayesian methods for subgroup analysis are illustrated through their use in analyzing the SOLVD data, and we compare and contrast the results of both the frequentist and Bayesian approaches. Connections between the data analysis and the capabilities of the **beanz** software are also emphasized throughout this section. Remarks regarding multiplicity issues in Bayesian subgroup analysis are made in Sect. 5, and Sect. 6 describes approaches for model comparison and model checking. We conclude in Sect. 7 with a few final remarks.

## Motivating example: the SOLVD trial

The studies of left ventricular dysfunction (SOLVD) described in The SOLVD Investigators (1991) examined the impact of the drug Enalapril in a group of patients with congestive heart failure and low ejection fraction. In total, 2569 patients were enrolled in the treatment trial with 1285 patients being assigned to the treatment arm and 1284 patients being assigned to the placebo arm. After the scheduled end of the study, 510 patients had died in the placebo group while 452 had died in the Enalapril group.

**Table 1:** Number of patients by treatment and subgroup covariates

Subgroup	Enalapril	Placebo	Total
Gender			
Female	259	244	503
Male	1025	1040	2065
Age			
≤65	866	862	1728
>65	418	422	840
Ejection fraction			
6–22	468	474	942
23–29	407	417	824
30–35	409	393	802

**Fig. 1:**
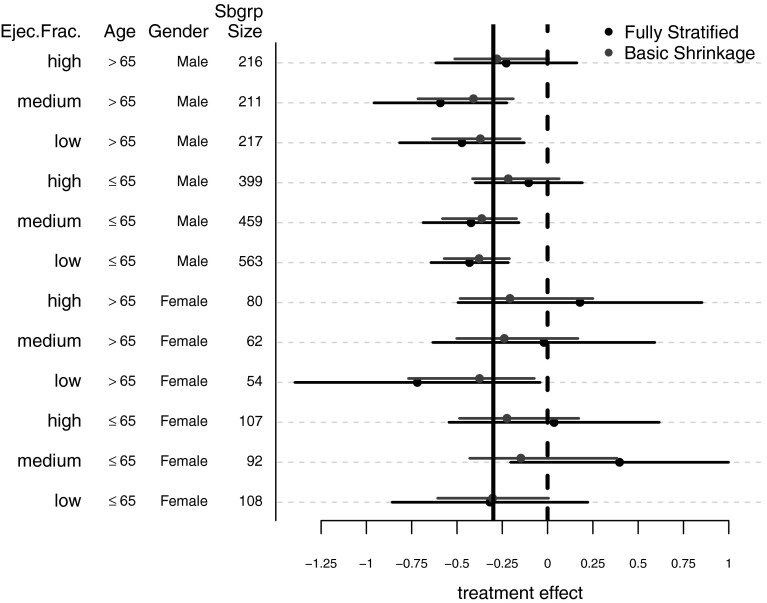
Basic shrinkage model. SOLVD data. Posterior means and frequentist estimates for each of the 12 subgroups defined by the variables gender, age, and ejection fraction. Frequentist estimates $\hat{\theta }_{g}$ and associated $95\,\%$ confidence intervals are in *black* while Bayes estimates and associated $95\,\%$ credible intervals are in *red*. The *solid vertical line* represents the estimated overall treatment effect from the basic shrinkage model, namely, the posterior mean of $\tau$ (Color figure online)

Due to the importance of ejection fraction in determining the target population, we examine response to treatment in subgroups defined by baseline ejection fraction, gender, and age. We dichotomized age into ≤65 and >65 years subgroups, and we discretized baseline ejection fraction by tertiles as was done in the original paper (The SOLVD Investigators 1991) describing this study. This way of discretizing age and ejection fraction yielded 12 subgroups in total. One patient was dropped from our analysis due to a missing ejection fraction value. Table 1 shows cross-tabulations for each of the variables used in the subgroup analysis, and Fig. 1 shows the number of patients within each of the 12 subgroups.

The original paper reporting on this study (The SOLVD Investigators 1991) concluded that the addition of Enalapril to standard therapy had a positive impact on patient mortality and hospitalization. Fitting a Cox-proportional hazard model using patients from the treatment trial supports this conclusion; the estimated log-hazard ratio of the Enalapril group to the control group was $-0.32$ with an associated standard error of 0.06. The survival endpoint used here and throughout the paper is time-to-death or hospitalization. While Enalapril appears to have had a beneficial overall effect in this trial, we are mainly interested in exploring any treatment effect heterogeneity in terms of key baseline covariates.

## Frequentist methods for subgroup analysis

### Univariate subgroup analysis

Univariate tests of interaction investigate each variable one-at-a-time to determine if there is an interaction between treatment and the specified variable. Because any such treatment interaction is an indication that the treatment effect varies across the levels of a subgroup variable, interaction tests are deemed important in assessing consistency of treatment effect across patient subgroups. Indeed, the FDA Guidance for Industry (see Food and Drug Administration 1998; Varadhan and Wang 2014) states that investigators provide evidence for “consistency across key patient subsets” in order to address concerns about the generalizability of trial results.

Suppose that for subjects $(1,\ldots ,n)$ we have observed continuous outcomes ${{\varvec{y}}}= (y_{1}, \ldots , y_{n} )$ and assigned treatments $T_{1},\ldots ,T_{n}$ with either $T_{i} = 0$ or $T_{i} = 1$. Suppose further that the $j$th covariate has *K*(*j*) levels and $X_{ijk}$ is an indicator of whether or not patient *i* has the $(k+1)^{st}$ level of the $j$th covariate. Then, in the following regression for the expected outcome

1 $$\begin{aligned} E(y_{i}| X_{ijk}, T_{i} ) = \beta _{0} + \gamma _{0}T_{i} + \sum _{k=1}^{K(j)-1} \beta _{k} X_{ijk} + T_{i} \sum _{k=1}^{K(j)-1} \gamma _{k} X_{ijk}, \end{aligned}$$

a univariate test of interaction (for the $j$th covariate) tests the null hypothesis $H_{0}: \gamma _{1} = \cdots = \gamma _{K(j)-1} = 0$. In other words, a univariate test of interaction (for the $j$th covariate) tests whether or not the effect of treatment is the same across all levels of the $j$th covariate. Analogous univariate tests may be performed for other types of responses such as binary or time-to-event outcomes.

To test for consistency of effect across the key identified patient subgroups in the SOLVD trial, we separately performed univariate tests of treatment interaction using the variables age, gender, and baseline ejection fraction. For each variable, we tested whether or not the variable–treatment interaction coefficients were all equal to zero or not. Because the outcomes in SOLVD are time-to-event, we used a Cox-proportional hazard models where the regression equations for the hypothesis tests were as in (1). These tests yielded *p*-values 0.40, 0.034, and 0.029 for age, gender, and ejection fraction respectively. At first glance, these results suggest there are treatment interactions with gender and ejection fraction which could raise doubts about the consistency of treatment effect. However, when performing a series of univariate tests, it is important to adjust for the multiplicity of tests (see, e.g. Varadhan and Wang 2014), and when using the Bonferroni adjustment for multiplicity, the adjusted *p* values for gender and ejection fraction were 0.102 and 0.087 respectively which considerably weakens the evidence for lack of consistency.

Despite their effectiveness in detecting the presence of interactions, univariate one-variable-at-a-time interaction tests have several limitations that restrict their usefulness in assessing treatment effect heterogeneity. Firstly, univariate interaction tests are not, in general, able to determine the direction or the magnitude of the treatment interaction of interest (see e.g., Alosh et al. 2015). When the variable in question has more than two levels, an interaction test can only lead to the conclusion that the treatment effects are not the same in all the subgroups but cannot detect the direction of treatment effect changes. Even when there are only two subgroup levels, interaction tests are not well-suited for assessing the magnitude of an interaction effect. Estimates of effect are often highly variable for small subgroups and are biased when only examined after they yield a significant result. As a result, it can be difficult to judge the importance of the treatment effect difference between two subgroups even when an interaction test yields a significant result.

**Table 2:** Cox proportional hazards model the covariates: treatment, age, gender, baseline ejection fraction, and the interactions between treatment and each of age, gender, and baseline ejection fraction

	Coefficient	SE	z-value	*p* value
trtment	−0.183	0.147	−1.242	0.214
age>65	0.251	0.078	3.225	0.001
genderMale	0.153	0.095	1.605	0.108
ejecfrac-medium	−0.291	0.087	−3.365	0.001
ejecfrac-high	−0.638	0.093	−6.894	<0.001
trtment:age>65	−0.121	0.116	−1.045	0.296
trtment:Male	−0.278	0.139	−1.998	0.046
trtment:ejecfrac-medium	0.112	0.129	0.868	0.386
trtment:ejecfrac-high	0.354	0.136	2.601	0.009

Estimated regression coefficients and corresponding standard errors are shown

### Unstructured interaction tests

An additional concern with univariate analyses is that they ignore the correlation among patient characteristics and examine each variable in isolation. As noted by Kent and Hayward (2007), a limitation of univariate subgroup analysis is that the univariate subgroups are less likely to identify important heterogeneity in treatment effect since the subgroups only differ in terms of a single characteristic. Overlooking such structure in the correlation among subgroups can result in confounding and other misleading inferences (Varadhan and Wang 2014). For example, suppose that the effectiveness of a drug varies by body weight independently of gender. In this case, an apparent marginal treatment–gender interaction would be largely driven by differences in treatment effectiveness by weight despite there being no difference in treatment response by gender when adjusting for body weight. This concern can be addressed by performing an unstructured interaction test (Kovalchik et al. 2013) where all patient covariates are included rather than focusing on one covariate at a time as is done in univariate analyses. In particular, using notation as in (1), an unstructured interaction test will test the null hypothesis that all $\gamma _{jk}$ equal zero in the following regression model for the expected outcome

2 $$\begin{aligned} E(y_{i}| X_{ijk}, T_{i} ) = \beta _{0} + \gamma _{0}T_{i} + \sum _{j=1}^{J}\sum _{k=1}^{K(j)-1} \beta _{jk} X_{ijk} + T_{i} \sum _{j=1}^{J}\sum _{k=1}^{K(j)-1} \gamma _{jk} X_{ijk}. \end{aligned}$$

In contrast to the univariate one-at-a-time approach, the full unstructured interaction approach tests whether or not there is at least one treatment–subgroup interaction when all of the other covariates are present in the model. However, as with univariate interaction tests, an unstructured interaction test can only determine if a treatment interaction is present, and a rejection of the test does not directly indicate which subgroups are the source of the interaction and does not characterize the magnitude of the subgroup treatment effect differences.

Using the SOLVD data with the same three covariates of age, gender, and ejection fraction, we performed a full, unstructured interaction test. For this test, we used a Cox-proportional hazards model with a regression formulation as in (2). This test yielded a *p* value of 0.018 suggesting that the treatment effect may not be constant across all subgroups. Summary output from a fit of the full interaction model is shown in Table 2.

A fully stratified subgroup analysis calculates treatment effects in each subgroup combination of the patient covariates. For example, if there are two covariates—gender (male/female) and age (young/old), then a fully stratified analysis reports the results for each of the four possible subgroups. While addressing some of the problems with univariate one-at-a-time analyses, fully stratified analyses will typically have much smaller subgroup-specific sample sizes and highly variable subgroup effect estimates.

## Bayesian methods for subgroup analysis

### Sampling model and notation

In the following description of subgroup analysis, we assume that summary statistics $(\hat{\theta }_{g}, s_{g}^{2})$ have been computed for each of the mutually exclusive subgroups $g = 1,\ldots ,G$. The estimates $\hat{\theta }_{g}$ are typically fully stratified frequentist estimates with the statistic $\hat{\theta }_{g}$ representing an estimate of the treatment effect in subgroup *g* and $s_{g}$ representing the standard error associated with $\hat{\theta }_{g}$. Reduction of data to the form $(\hat{\theta }_{g}, s_{g}^{2})$ includes a wide range of possible settings including, for example, cases where $\hat{\theta }_{g}$ is a difference of treatment means, cases where $\hat{\theta }_{g}$ represents an estimated log-odds ratio, or cases where $\hat{\theta }_{g}$ is an estimated log-hazard ratio. The **beanz** web-based software tool allows the user to either input subgroup-level summary statistics $(\hat{\theta }_{g}, s_{g}^{2})$ or to input the subject-level raw data, in which case **beanz** computes the necessary summary statistics for each subgroup.

Often, $\hat{\theta }_{g}$ may be interpreted as a maximum likelihood estimate of the underlying treatment effect of interest $\theta _{g}$, and as such, standard statistical theory asserts that a good approximation to the sampling distribution of $\hat{\theta }_{g}$ is

3 $$\begin{aligned} \hat{\theta }_{g}|\theta _{g} \sim {\text {Normal}}\left(\theta _{g}, s_{g}^{2}\right). \end{aligned}$$

One criticism of (3) is that it ignores the uncertainty about the variance of $\hat{\theta }_{g}$ and simply plugs-in an estimate $s_{g}^{2}$ of this variance. However, in the absence of additional information beyond $(\hat{\theta }_{g}, s_{g}^{2})$, (3) serves as a suitable approximation in most cases and may be preferable to inappropriate modeling of the uncertainty associated with $s_{g}$. Indeed, several authors including Jones et al. (2011) suggest using the sampling distribution in (3) as a reasonable approximation.

For the SOLVD data, we define the subgroup treatment effect $\theta _{g}$ as the log-hazard ratio between the treatment (Enalapril) group and the placebo group which implies that smaller treatment effects correspond to greater treatment benefit. The treatment effect estimates $\hat{\theta }_{g}$ and associated standard errors $s_{g}$ were computed by fitting a Cox-proportional hazards model within each of the $G = 12$ subgroups. The estimated treatment effect $\hat{\theta }_{g}$ is the estimated log-hazard ratio between the Enalapril and placebo groups. It is worth mentioning that the treatment effect estimates $\hat{\theta }_{g}$ were computed using time-to-death or hospitalization as the outcome while the example on the **beanz** website involving the SOLVD data currently uses time-to-death or hospitalization after 1 year of follow-up as the outcome. In **beanz**, when the responses are time-to-event and when the user inputs the original, subject-level data rather than subgroup-level summary statistics, **beanz** computes estimated log-hazard ratios $\hat{\theta }_{g}$ and standard errors $s_{g}$ by fitting a Cox-proportional hazards model within each of the defined subgroups.

### The basic shrinkage model

#### Model description

The basic shrinkage model is a general approach for analyzing variation in treatment effect. This model is employed in a wide range of applications and is particularly suited to settings such as subgroup analysis where one is interested in separate units of analysis with each unit having a relatively small sample size. Its effectiveness in these settings is largely due to the “partial-pooling” or “shrinkage” phenomenon where individual subgroup effect estimates are shrunken towards an overall global mean. Despite its reliance on the often implausible assumption of full exchangeability, the basic shrinkage model is a useful starting point because it illustrates many recurring themes of Bayesian analysis and because it frequently serves as an effective and robust approximation. Moreover, from the perspective of analyzing HTE, the exchangeability assumption serves as a reasonable a-priori position since it does not make any a-priori distinctions among the subgroup-level treatment effects.

The basic shrinkage model starts with the sampling model of (3) for the observed effect estimates $\hat{\theta }_{g}$ and adds the assumption that the underlying subgroup treatment effects $\theta _{g}$ are drawn from a common normal distribution with mean $\tau$ and standard deviation $\omega$. A fully hierarchical approach places a prior on the hyperparameters $\tau$ and $\omega$, and as in Jones et al. (2011), we suggest a normal prior for $\tau$ and a half-normal prior for $\omega$ in order to complete the specification of the model

4 $$\begin{aligned} \theta _{g}|\tau , \omega&\sim {\text {Normal}}(\tau , \omega ^{2}) \nonumber \\ \tau&\sim {\text {Normal}}(0, \sigma _{\tau }^{2}) \nonumber \\ \omega&\sim {\text {Half-Normal}}(\sigma _{\omega }^{2}). \end{aligned}$$

In the above, the parameter $\tau$ should be thought of as the true overall or average treatment effect and $\omega$ should be thought of as the standard deviation of the treatment effect across subgroups. Thus, larger values of $\omega$ indicate greater treatment effect heterogeneity. When $\omega$ is treated as a fixed quantity, (4) is commonly referred to as a one-way random effects model. Models similar to (4) are popular in random-effects meta-analysis (see e.g., Sutton and Abrams 2001) where the parameters $\theta _{g}$ are thought of as study-specific treatment effects drawn from a common population distribution, which in the case of model (4) is assumed to be ${\text {Normal}}(\tau , \omega ^{2})$. In the absence of substantial prior information regarding possible values of $\tau$ and $\omega$, it is recommended to start with diffuse priors for $\tau$ and $\omega$ (i.e., large $\sigma _{\tau }^{2}$ and $\sigma _{\omega }^{2}$) in order to accommodate a wide range of values for both the overall treatment effect and the variation across subgroups.

In practice, the basic shrinkage model can be thought of as providing a compromise between the fully stratified, separate analysis described at the end of Sect. 3 and a completely pooled analysis which ignores any differences among subgroups and only reports the overall response to treatment. In contrast to these two extremes, estimates of the within-subgroup treatment effects in the basic shrinkage model are determined by both the fully stratified estimate $\hat{\theta }_{g}$ and the overall treatment effect estimate $\hat{\tau }$. In particular, in the basic shrinkage model, each estimate of the subgroup-level treatment effect is a weighted average of the original subgroup-specific estimate $\hat{\theta }_{g}$ and the global treatment effect estimate which causes each Bayesian estimate to be pulled or “shrunken” towards the estimated overall treatment effect. This shrinkage phenomenon may be more clearly observed by looking at the form of the posterior mean of $\theta _{g}$

5 $$\begin{aligned} E\left( \theta _{g} | {{\varvec{y}}}\right) = \hat{\tau } + E\left(\left.\frac{ \omega ^{2} }{ \omega ^{2} + s_{g}^{2} } \right| {{\varvec{y}}}\right )\left( \hat{\theta }_{g} - \hat{\tau } \right) , \end{aligned}$$

where $\hat{\tau } = E(\tau |{{\varvec{y}}})$ is the posterior mean of the overall treatment effect. In other words, $E(\theta _{g}| {{\varvec{y}}})$ is equal to the overall treatment effect estimate plus a proportion of the distance between $\hat{\theta }_{g}$ and $\hat{\tau }$. The posterior mean of the fraction $r(\omega , s_{g}) = \omega ^{2}/(\omega ^{2} + s_{g}^{2})$ determines the magnitude of shrinkage with $E( r(\omega , s_{g}) | {{\varvec{y}}}) = 0$ implying complete shrinkage to the overall treatment effect and $E\left( r(\omega , s_{g}) | {{\varvec{y}}}\right) = 1$ implying no shrinkage at all. The fact that $r(\omega , s_{g})$ increases with $\omega$ and decreases with $s_{g}$ has two important consequences: subgroups with high estimation variance (i.e., higher $s_{g}$) are shrunk more severely than subgroups with low estimation variance, and small values of $\omega$ are associated with stronger overall shrinkage effects. The greater shrinkage for those subgroups with higher estimation error is a reflection of the association between data sparsity and shrinkage effects. That is, subgroups with large sample sizes and consequent estimation precision are treated as more reliable estimates and are not shrunken much towards the overall effect estimate. Turning to the role of $\omega$, because small values of $\omega$ indicate little heterogeneity in treatment effect it should not be surprising that selecting a prior for $\omega$ with most of the probability near zero will induce greater shrinkage across subgroups resulting in tighter clustering of subgroup effects near $\hat{\tau }$. Indeed, setting a prior for $\omega$ which is concentrated near zero is an effective approach for expressing prior skepticism regarding the presence of treatment effect heterogeneity.

The shrinkage phenomenon and information sharing across subgroups arises from the shared normal distribution of the underlying treatment effects $\theta _{g}$. Because this distribution acts as a kind of common prior for each specific subgroup effect $\theta _{g}$, this enables an estimate for a specific subgroup *g* to partly “learn” from the evidence provided by the outcomes in the other subgroups rather than only using the data from subgroup *g*. For instance, if the majority of other subgroup effect estimates are tightly centered around $\hat{\tau }$, larger values of $\hat{\theta }_{g}$ will tend to be pulled back towards the overall estimate because the evidence from the other subgroups suggests there is very little heterogeneity in treatment effect. This information sharing has the consequence of dampening the more extreme subgroup outcomes that often occur when there are small within-subgroup sample sizes.

Figure 1 presents an application of the basic shrinkage model to the 12 subgroups from the SOLVD trial. The figure shows, for each of the 12 subgroups, estimates $\hat{\theta }_{g}$ of the log-hazard ratio between two treatments of interest along with the corresponding posterior means and $95\,\%$ credible intervals obtained from the basic shrinkage model. The solid vertical line placed at $-0.30$ in Fig. 1 corresponds to the posterior mean $\hat{\tau }$ of $\tau$ from the basic shrinkage model. The estimate $\hat{\tau }$ can be interpreted as the estimated overall treatment effect from the basic shrinkage model, and as is usually the case, this estimate $\hat{\tau } = -0.30$ is quite close to the overall treatment effect estimate of $-0.32$ obtained from fitting a Cox proportional hazards model without any adjustment for baseline covariates. In addition to nicely demonstrating the shrinkage of the Bayes estimates towards the estimated overall treatment effect, Fig. 1 shows the usual greater precision of the Bayes estimates compared to the raw, un-shrunken treatment effect estimates. Another feature of Fig. 1 worth highlighting is the differential shrinkage across subgroups where, due to higher estimation variance, treatment effect estimates for subgroups with fewer numbers of patients tend to be shrunk more strongly. Differential shrinkage can sometimes result in cases where a ranking of the Bayes estimates is different than that of the original frequentist estimates. In fact, an example of this may be seen in Fig. 1 by comparing the (low ejec.frac./age >65/female) subgroup and the (medium ejec.frac./age >65/male) subgroup. The original treatment effect estimate for the (low ejec.frac./age >65/female) subgroup is more extreme than that of the (medium ejec.frac./age >65/male) subgroup, but the greater shrinkage of the highly variable (low ejec.frac./age >65/female) subgroup estimate results in the Bayes estimate for the (low ejec.frac./age >65/female) subgroup being closer to $\hat{\tau }$ than the Bayes estimate for the (medium ejec.frac./age >65/male) subgroup. The **beanz** software tool automatically generates forest plots similar to Fig. 1 for each of the Bayesian models that the user chooses to fit.

Overall, Fig. 1 demonstrates substantial shrinkage of the fully stratified frequentist estimates with particularly strong shrinkage for the female subgroups. With this strong shrinkage towards the overall treatment effect there appears to be little evidence of particular subgroups that have a substantially different response to treatment. There does, however, appear to be greater treatment effectiveness in men versus women and in subgroups with lower baseline ejection fraction. In addition to looking at the variation in treatment effect across subgroups, another key question is whether or not there are subgroups where the treatment effect has a different sign than the overall effect. On this issue, there is little evidence from the basic shrinkage model of such qualitative interactions. All of the posterior means are less than zero with the most modest estimated treatment effect of $-0.12$ occurring in the (medium ejec.frac./age ≤65/female) subgroup. This stands in contrast to the frequentist estimates where the overall picture is not quite as coherent. Three of the frequentist point estimates are positive, and one of the subgroup confidence intervals does not cover the overall treatment effect of $-0.30$. Although the extreme point estimates for subgroups (high ejec.frac./age >65/female) and (medium ejec.frac./age ≤65/female) seem to point at interesting subgroup effects, such results should not be especially surprising when analyzing a collection of highly variable estimates. The variation exhibited by the un-shrunken estimates is usually much greater than the variation of the underlying true treatment effects, and extreme values of the un-shrunken estimates should be viewed with considerable skepticism. Subgroups with small numbers of patients tend to have the highest variance, and indeed, as shown in Fig. 1, the two previously highlighted subgroups with extreme point estimates of treatment effect [i.e., the (high ejec.frac./age >65/female) and (medium ejec.frac./age ≤65/female) subgroups] are among the smallest subgroups of the 12 subgroups.

#### Role of priors and sensitivity analysis

**Fig. 2:**
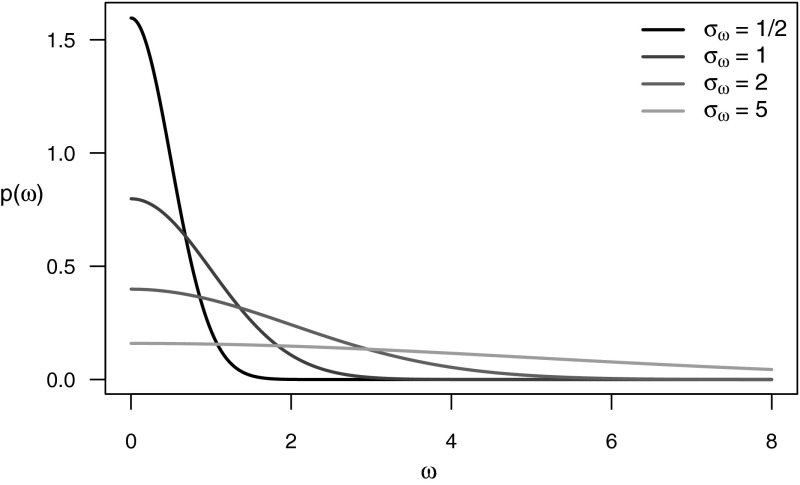
Half-normal densities plotted for several values of the scale parameter: $\sigma _{\omega } = 1/2$, $\sigma _{\omega }=1$, $\sigma _{\omega } = 2$, and $\sigma _{\omega } = 5$

Implementation of the basic shrinkage model requires a choice of the hyperparameters $\sigma _{\tau }^{2}$ and $\sigma _{\omega }^{2}$ which refer to the priors for the overall treatment effect and the variation in subgroup-specific treatment effect respectively. As a sensible default choice, we recommended using “non-informative” priors for both $\tau$ and $\omega$ so that posterior inferences are not unduly influenced by information not contained in the data being analyzed. Non-informative priors are often implemented by choosing a vague or diffuse proper prior that spreads the prior evenly over a broad range of possible values. For the overall treatment effect $\tau$, one can specify a diffuse prior by choosing a large value of $\sigma _{\tau }^{2}$ such as $\sigma _{\tau }^{2} = 10^{6}$ recommended in Jones et al. (2011) or $\sigma _{\tau }^{2} = 10^{3}$ as is the default setting in **beanz**. As stated in Eq. (4), the standard deviation of treatment effect across subgroups is determined by the parameter $\omega$ which is assigned a half-normal prior with parameter $\sigma _{\omega }^{2}$. To assess the plausibility of different values of $\sigma _{\omega }^{2}$, it is helpful to recall the definition of a half-normal distribution. The random variable *Y* is said to have a half-normal distribution with parameter $b^{2}$ (i.e., $Y \sim {\text {Half-Normal}}(b^{2}$) ) if *Y* has the same distribution as *b*|*Z*| where *Z* is a standard normal random variable. This definition implies the median of the prior for $\omega$ is $0.674 \times \sigma _{\omega }$, the 75th percentile is $1.150 \times \sigma _{\omega }$, and the 99th percentile is $2.576 \times \sigma _{\omega }$. Densities of half-normal distributions for several values of $\sigma _{\omega }$ are shown in Fig. 2.

In the context of selecting an approximately non-informative half-normal prior for the random effects standard deviation $\omega$ when the treatment effects are mean differences, Gelman (2006) suggests setting $\sigma _{\omega } = 100$. In other contexts where the parameters of interest are on a different scale, it may be sensible to use a more informative prior. As noted by Spiegelhalter et al. (2003) and others, when $\theta _{g}$ represents a log-odds ratio setting $\sigma _{\omega } = 1$ is a reasonable choice since a standard deviation greater than three in the log-odds ratio across subgroups would be considered quite large in almost any context. The half-normal prior with $\sigma _{\omega } = 1$ can then be viewed as a weakly informative prior in the sense that it is does not utilize any prior information specific to the problem at hand but only uses what is known about the scale to construct a prior which gives most of its weight to all the plausible values on this scale. Using the half-normal prior rather than more traditional non-informative priors can have an impact on posterior inferences especially for cases when there are few subgroups or for cases when the variation in treatment effect across subgroups is close to zero. In any setting, it is important to think about the scale on which the outcome is measured and the interpretation of the corresponding parameters and priors. As the default settings of $\sigma _{\tau }^{2}$ and $\sigma _{\omega }^{2}$ in the basic shrinkage model, **beanz** uses $\sigma _{\tau }^{2} = 1000$ and $\sigma _{\omega }^{2} = 100$, but these values of the hyperparameters can be adjusted by the user in the configuration panel of the **beanz** software tool.

Regardless of the choice of prior, it is advisable to conduct some type of sensitivity analysis to examine the impact of changing the prior or other features of the model on posterior inferences of interest. In the context of the basic shrinkage model, one way to investigate the sensitivity of the results is to consider a range of values for the hyperparameter $\sigma _{\omega }$ and compute posterior quantities such as subgroup-specific posterior means and posterior quantiles for each value of $\sigma _{\omega }$. In this case, no practical changes in the main inferences is an indication of robustness of these inferences to prior specification. If some of the posterior inferences do change substantively in a sensitivity analysis, fitting the model for several different priors will still enable one to analyze and report which conclusions are dependent on which features of the prior. One can then use this information to further evaluate the strength of any claimed effect; for instance, reporting that a subgroup seems to have a positive treatment effect except when using a highly skeptical prior for $\tau$ may be a useful conclusion and worth reporting. Here, a skeptical prior (see e.g., Spiegelhalter et al. 2003) for the overall treatment effect refers to a prior which is heavily concentrated near zero and where large treatment effects are viewed skeptically and hence given small prior probability.

As discussed in Sect. 4.2.1, the basic shrinkage model results shown in Fig. 1 provide evidence against any substantial differences across subgroups though there is perhaps some evidence of reduced treatment benefit in women and in those with higher ejection fraction levels. To probe the sensitivity of these conclusions to sensible changes in the model, we fit the basic shrinkage model for several different choices for the distribution of the variance component $\omega$. We focused on the distribution of $\omega$ because the choice of the variance component distribution often has a meaningful impact on posterior inferences particularly when there are few subgroups and/or when the variation in treatment effect is close to zero.

**Fig. 3:**
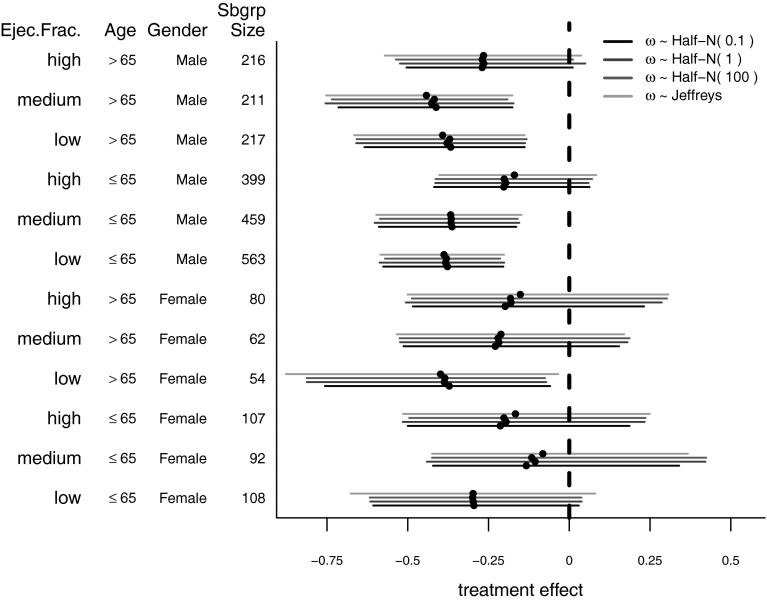
Basic shrinkage model—sensitivity to choice of prior. SOLVD data. Posterior means and associated credible intervals for the following choices of the prior for $\omega$: $\omega \sim {\text {Half-Normal}}( 0.1 )$, $\omega \sim {\text {Half-Normal}}( 1 )$, $\omega \sim {\text {Half-Normal}}( 100 )$, and $\omega \sim {\text {Jeffreys}}$. The approximate Jeffreys prior for $\omega ^{2}$ employed here is $p(\omega ^{2}) \propto \omega ^{-2}$ for $\omega ^{2} \ge 0.005$ and $p(\omega ^{2}) = 200$ otherwise

When varying the distribution of $\omega$ for this sensitivity analysis, we used several different half-normal distributions along with the approximate Jeffreys prior suggested by Dixon and Simon (1991). Posterior means and associated credible intervals are shown in Fig. 3. Changing the value of $\sigma _{\omega }$ in the half-normal distribution seems to have very little impact even though this parameter was varied from 0.1 to 100. Using an approximate Jeffreys prior for $\omega$ also does not seem to have much of an impact when compared to the half-normal priors. With regard to the approximate Jeffreys prior, it is worth mentioning that there seems to be moderate sensitivity to the choice of the truncation point (i.e., 0.005 in the caption of Fig. 3) and that only the results for the truncation point suggested by Dixon and Simon (1991) are shown in Fig. 3. The approximate Jeffreys prior suggested by Dixon and Simon (1991) is used here because the true Jeffreys prior in this context is an improper prior. In this case study, we have examined 12 subgroups. Posterior inferences will likely be more sensitive to the prior for $\omega$ when there are fewer subgroups because, in these case, there is not much information regarding the variation in treatment effect across subgroups.

Embedded in the basic shrinkage model is the assumption of exchangeability of the subgroup treatment effects. This means that, a-priori, there is no reason to favor any specific subgroup or to group any collection of subgroups in a particular way. Essentially, the exchangeability assumption is one that must be made if the subgroups were simply labeled $1,\ldots ,G$ without any further information about the covariates which make up the subgroups. Exchangeability is often a reasonable assumption, for example, when the subgroups are defined by the levels of one variable and there is no prior information suggesting that the treatment effects will be larger in any specific subgroup. In other contexts, exchangeability may be seen as simply a reasonable working assumption. More flexible modeling which relaxes the assumption of exchangeability can be done by employing regression models for the subgroup treatment effects, which are discussed in the following subsection.

### Regression models

#### Dixon–Simon

Compared to the basic shrinkage model, regression models for the treatment effects offer a more flexible way to represent the influence of each variable on the subgroup-specific treatment effects and to capture the relationships among the subgroups. One of the most straightforward regression models assumes that each subgroup treatment effect can be expressed as a linear combination of individual variable effects. In particular, the relationship between the subgroup treatment effect $\theta _{g}$ and the variables that compose subgroup *g* is modeled as

6 $$\begin{aligned} \theta _{g} = \tau + \sum _{j=1}^{J}\sum _{k=1}^{K(j)-1} X_{gjk}\beta _{jk}, \end{aligned}$$

where $K(j) \ge 2$ is the number of levels of variable *j* and where $X_{gjk} = 1$ when the $(k+1)^{st}$ level of variable *j* belongs to subgroup *g* and $X_{gjk} = 0$ otherwise. In the parameterization of (6), $\tau$ no longer represents the overall treatment effect but, rather, represents the treatment effect in the subgroup where each variable has the reference level (i.e., level 1). The regression coefficient $\beta _{jk}$ should then be interpreted as the change in the treatment effect when variable *j* moves from the reference level to level $k + 1$. For the SOLVD data, the reference level used for the gender variable was female; the reference level used for the age variable was age ≤65; and the reference level used for the ejection fraction variable was the high ejection fraction category.

In the context of using (6) for subgroup analysis, Dixon and Simon (1991) suggest basing the prior on the assumption that the regression coefficients are drawn from a common normal distribution with mean zero and standard deviation $\omega$, and the authors assign $\omega ^{2}$ the approximate non-informative Jeffreys prior $p(\omega ^{2}) \propto [\max \{ \omega ^{2}, \varepsilon \}]^{-1}$, where $\varepsilon$ is a small, positive quantity. If, as in Jones et al. (2011), we replace the Jeffreys prior for $\omega$ with a half-normal distribution, then the modified Dixon–Simon model has the following structure

$$\begin{aligned} \tau&\sim {\text {Normal}}(0, \sigma _{\tau }^{2}) \\ \beta _{jk}&\sim {\text {Normal}}(0, \omega ^{2}) \\ \omega&\sim {} {\text {Half-Normal}}(\sigma _{\omega }^{2}). \end{aligned}$$

In comparison to estimates of $\beta _{jk}$ computed in the classical approach to regression, the shared distribution of the regression coefficients in the Dixon–Simon specification shrinks the posterior means of $\beta _{jk}$ towards zero. In addition to inducing desirable shrinkage of subgroup treatment effects, the regression model (6) is able to better account for the correlation that exists among subgroups than the basic shrinkage model. For example, suppose we have subgroups defined by the two variables gender (male/female) and age (young/old). If we consider subgroup *g* (female/young) and subgroup $g'$ (female/old), the treatment effects for these two subgroups using the regression model (6) would be

$$\begin{aligned} \theta _{g}&= \tau + \beta _{11} \\ \theta _{g'}& = {} \tau + \beta _{11} + \beta _{21}. \end{aligned}$$

Correlation between these two subgroups is induced through the shared intercept $\tau$ and shared gender coefficient $\beta _{11}$. Moreover, the regression structure implies that subgroups which are “further apart” have less correlation than more closely related subgroups. For instance, the prior correlation between the subgroups (female/young) and (female/old) is greater than that between subgroups (female/young) and (male/old) since the former pair share the coefficients $(\tau ,\beta _{11})$ while the latter pair only share the intercept term. This richer correlation structure of the Dixon–Simon model stands in contrast to the basic shrinkage model which models the correlation equally across all subgroups. As in the basic shrinkage model, we suggest using diffuse priors as the default choices for both the intercept $\tau$ and the regression coefficients. This can be done by choosing relatively large values of $\sigma _{\tau }^{2}$ and $\sigma _{\omega }^{2}$ such as 1000 and 100 respectively.

#### Other regression models and the extended Dixon–Simon model

A number of other regression models for subgroup analysis have been suggested in Jones et al. (2011) and implemented in the **beanz** software tool. These include a basic regression model which places diffuse priors on the coefficients without a shared variance component, and a basic regression and shrinkage model which adds a regression component to the basic shrinkage model. Descriptions of these additional regression models are provided in Wang et al. (2016).

In addition to these, **beanz** implements an extension of the Dixon–Simon model suggested in Jones et al. (2011) which allows for higher orders of interaction between the patient covariates. In the case of three covariates with two levels each, for example, the extended Dixon–Simon model for the treatment effects is

7 $$\begin{aligned} \theta _{g} = \tau + \sum _{j=1}^{3} X_{gj1}\beta _{j1} + \sum _{j=1}^{2} \sum _{h=j+1}^{J} \gamma _{jh} X_{gj1}X_{gh1} + \delta X_{g11}X_{g21}X_{g31}. \end{aligned}$$

The regression coefficients for each order of interaction in (7) are assigned a Normal distribution with a common variance. In particular, the priors for the model parameters in (7) are $\tau \sim N(0, \sigma _{\tau }^{2})$, $\beta _{j1}|\omega _{1} \sim N(0,\omega _{1}^{2})$, $\gamma _{jh}|\omega _{2} \sim N(0, \omega _{2}^{2})$, and $\delta |\omega _{3} \sim N(0,\omega _{3}^{2})$. The variance components $\omega _{1},\omega _{2},\omega _{3}$ are assigned independent half-normal priors.

**Fig. 4:**
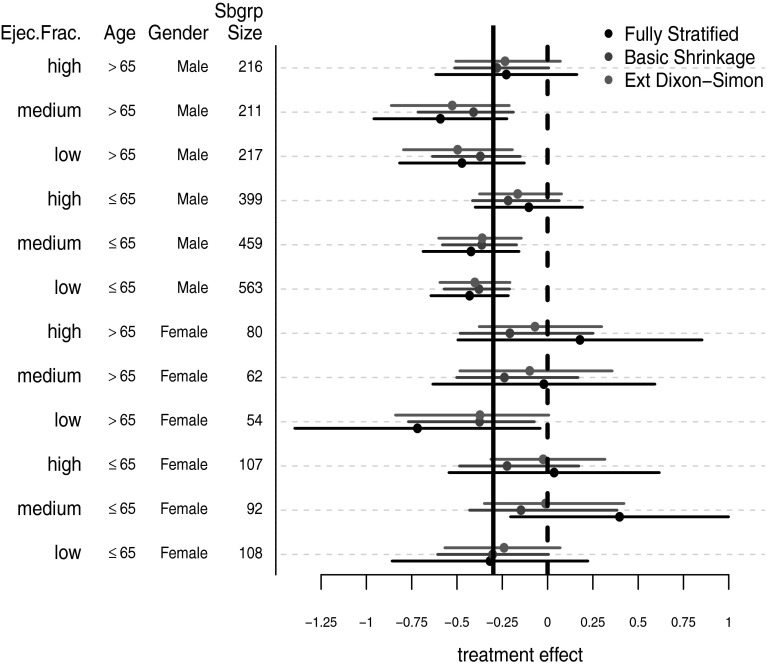
Extended Dixon–Simon model. SOLVD data. Posterior means and credible intervals for each of the 12 subgroups defined by the variables: gender, age, and baseline ejection fraction. Point estimates and uncertainty intervals from the basic shrinkage model and from the fully stratified frequentist analysis are also shown

Figure 4 shows posterior means and $95\,\%$ credible intervals obtained from applying the extended Dixon–Simon model [Eq. (7)] to the SOLVD data. Quantities from the basic shrinkage model along with the fully stratified frequentist estimates are also displayed in Fig. 4. It can be seen from this figure that the treatment effect estimates from the extended Dixon–Simon model exhibit somewhat more variability than the estimates from the basic shrinkage model. The most notable difference between the two Bayesian models in Fig. 4 is the reduced treatment effectiveness in women with medium or high ejection fraction levels suggested by the extended Dixon–Simon model. This is because, whereas the basic shrinkage model treats the subgroup categories as completely exchangeable labels, the regression structure of the extended Dixon–Simon model is able to better recognize that subgroups with higher ejection fraction levels consistently tend to respond worse to treatment and that male subgroups consistently respond better to treatment.

## Multiple comparisons

A chief concern in subgroup analysis is the multiple comparisons that arise from the various subgroups being considered. When using conventional approaches to hypothesis testing, the probability of falsely rejecting at least one of the no-interaction hypotheses grows as the number of subgroups increases, and failure to take this multiplicity into account can result in high error rates. Traditional approaches to handling multiplicity include reporting Bonferroni-adjusted *p*-values for each test performed, or adjustments that increase confidence interval widths. Despite their role in controlling Type I errors, such multiplicity adjustments can result in increased Type II errors and loss of power.

Tests of point hypotheses such as those discussed in Sect. 3 that test whether or not specific interaction terms are exactly equal to zero are not applicable within the context of the Bayesian models discussed thus far. The supposition in these models is that there is variation in treatment effect across subgroups, and they assign zero probability to the hypothesis that the parameters equal any particular value. Nevertheless, there may be concern about making certain erroneous statements regarding subgroup differences; for example, claiming that a subgroup-specific treatment effect is positive when the true treatment effect is actually negative.

In the context of the basic shrinkage model, making explicit adjustments for multiplicity is usually not necessary. Rather, one can just compare the individual credible intervals with the threshold of interest without taking into account the number of subgroups considered in the analysis. This is because shrinkage of more highly variable subgroup estimates tends to not only produce more reasonable point estimates of treatment effect but also prevents more “false positive” cases. As noted in Gelman (2006), Bayes procedures from hierarchical models that make no explicit multiplicity adjustments typically tend to be quite conservative when compared to their classical counterparts. This conservatism of Bayes estimates is exhibited in Fig. 1 where one can note that the posterior means from the basic shrinkage model tend to not deviate much from the estimated overall treatment effect. Because such automatic multiplicity adjustments arise from the joint modeling of the subgroup effects, such claims are dependent upon a well-calibrated model, and one should perform checks to ensure that the model used is justifiable. Approaches for model checking are discussed in the next section.

## Model choice and diagnostics

Factors to consider when selecting a model include: goodness-of-fit measures, model complexity, interpretability, plausibility of model assumptions, and scientific knowledge, among other possible considerations. Measures of predictive accuracy usually incorporate both goodness-of-fit and model complexity. The deviance information criterion (DIC) suggested by Spiegelhalter et al. (2011) is a measure of predictive performance analogous to the widely used Akaike information criterion (AIC) and can be easily computed for a wide range of Bayesian models. An attractive feature of DIC is that it can be used to compare models with widely different structure and complexity where the number of model parameters does not have a clear meaning. As with the AIC, the DIC is based on combining a measure of goodness-of-fit with a penalty term for model complexity. For the sampling distribution (3) assumed by **beanz**, the goodness-of-fit is captured by the posterior expected deviance $\bar{D} = E\left( D(\theta _{1},\ldots ,\theta _{G})| \mathbf {y} \right)$ where

$$\begin{aligned} D(\theta _{1},\ldots ,\theta _{G}) = \sum _{g=1}^{G}\Big ( \frac{ \hat{\theta }_{g} - \theta _{g} }{ s_{g} } \Big )^{2}, \end{aligned}$$

is a measure of deviance. The “effective number of parameters”, $p_{D}$, is defined as the difference $p_{D} = \bar{D} - D(\bar{\theta }_{1}, \ldots , \bar{\theta }_{G})$ where $\bar{\theta }_{g} = E(\theta _{g}|\mathbf {y})$ is the posterior mean of the subgroup treatment effect $\theta _{g}$. The effective number of parameters can be directly computed in a wide range of hierarchical models such as the basic shrinkage model where the number of parameters does not have a clear, unambiguous definition. The DIC is then defined as the posterior expected deviance plus the effective number of parameters

$$\begin{aligned} \text {DIC} = \overline{D} + p_{D} = 2\overline{D} - D(\overline{\theta }_{1}, \ldots , \overline{\theta }_{G}). \end{aligned}$$

Lower values of the DIC imply better measures of fit. A rough rule-of-thumb suggested by Carlin and Louis (2009) is that meaningful differences between DIC values start at differences of greater than three to five.

In total, **beanz** currently offers seven different Bayesian models for subgroup analysis. Of these seven different models, the extended Dixon–Simon model had the lowest value of the DIC when looking at the SOLVD data with the 12 subgroups defined by age, gender and ejection fraction. The difference in DIC between the basic shrinkage model and the extended Dixon–Simon model was 2.56. Though the extended Dixon–Simon is best in terms of DIC, this relatively small difference in DIC suggests that both the basic shrinkage and extended Dixon–Simon models could be justified as model choices.

While DIC is a useful tool for model comparison, the DIC alone is not necessarily helpful in checking whether a particular model provides a good fit to the data. Posterior predictive checks (Rubin 1984 or Gelman 2003) are a useful tool for checking the plausibility of a model and for uncovering particular features of the observed data that are not captured well by the model under consideration. In a nutshell, posterior predictive checks are performed by comparing hypothetical data generated from the fitted model with the observed data. If the posterior provides a good fit, one should expect samples from the posterior predictive distribution to resemble the observed data, or at least, one should not expect there to be systematic discrepancies between the posterior predictive distribution and the observed data. Posterior predictive checks are often carried out by choosing a particular test statistic $T({{\varvec{y}}})$ or a collection of test statistics and comparing the posterior predictive distribution of $T({{\varvec{y}}})$ (usually denoted as $p\left( T({{\varvec{y}}}^{rep})| {{\varvec{y}}}\right)$) with the observed value of $T({{\varvec{y}}})$. Samples from the posterior predictive distribution can be used to visually assess whether or not the observed value of the statistic $T({{\varvec{y}}})$ seems typical of hypothetical replications from the fitted model. More formal testing approaches involving the computation of posterior predictive *p* values have been suggested by others, for example, Meng (1994).

**Table:** Summary of key factors to consider when using Bayesian methods to analyze heterogeneity of treatment effects

**Fig. 5:**
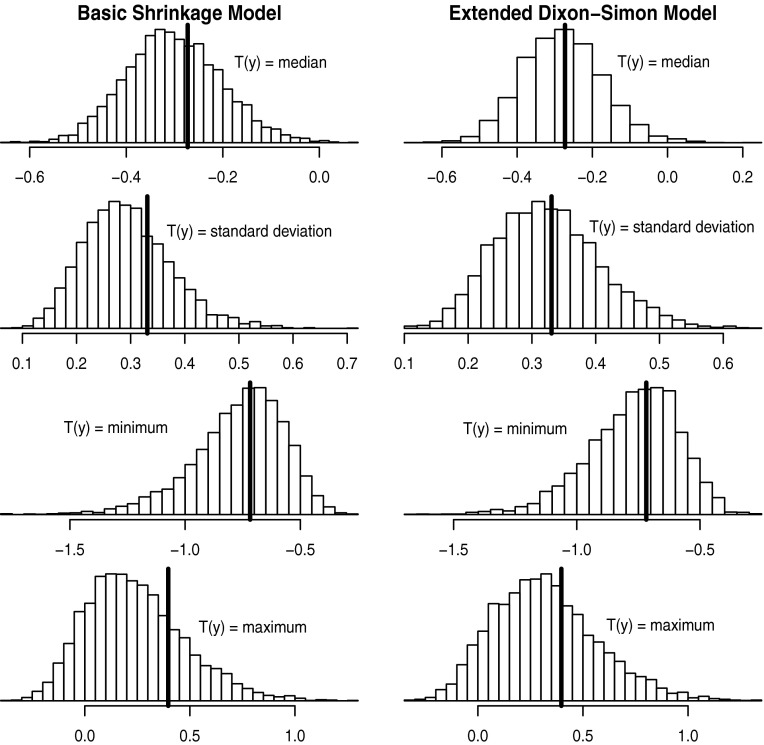
Posterior predictive checks. Samples from the posterior predictive distribution using both the basic shrinkage model and the extended Dixon–Simon model on the SOLVD data. Samples from the posterior predictive distribution of various test statistics *T*(*y*) are shown: median, standard deviation, minimum, and maximum. For each *panel*, the *solid vertical line* represents the observed value of the statistic *T*(*y*)

*Model specification*
The models described in Jones et al. (2011) and implemented by **beanz** offer a number of useful options for subgroup analysis. Parameters for the prior distributions can be chosen using common default values such as those used in the **beanz** software, through incorporating external information, or through knowledge about the scale of the outcome. In all cases, one should ensure that interpretations of the model parameters are well-understood and that the choice of priors is defensible.
*Diagnostics and model criticism*
If multiple models are considered, the deviance information criterion (DIC) can be used to compare model performance. Small differences (less than 3–5) in DIC are often not considered meaningful. Sensitivity analyses should be conducted by investigating changes in key posterior quantities over a range of different priors. Looking at posterior inferences in other plausible alternative models can also serve as a way of examining the sensitivity of the results. Posterior predictive simulations as depicted in Fig. 5 are a useful tool for checking if replicated data sets from the fitted model seem plausible in light of the observed data. Notable differences between posterior predictive simulations and the observed data suggest that one should consider modifying the model.
*Reporting and interpreting results*
Reporting posterior summaries for all subgroup parameters is often effective for characterizing HTE and for interpreting particular subgroups effects. Forest plots such as those shown in Figs. 1 and 4 are an effective way to visually represent this information. Posterior summaries related to many questions of clinical interest can usually be obtained from the full posterior distribution. For instance, an important question to consider is often whether or not there are qualitative interactions; that is, are there subgroups whose treatment effect is in the opposite direction of the average treatment effect? Finally, for full transparency, one should describe all steps taken in the analysis; for example, one should describe any changes made to the model during the course of the analysis, or if multiple models were originally entertained, one should describe why the final model was chosen.

In the context of the models discussed Sect. 4 where the sampling distribution is assumed by (3), one can generate a sample $\hat{\theta }_{g}^{pred,s}$ of subgroup effects from the posterior predictive distribution by first drawing $\theta _{g}^{post,s} \sim p(\theta _{g}|{{\varvec{y}}})$ from the posterior distribution of $\theta _{g}$ and then sampling $\hat{\theta }_{g}^{pred,s} \sim {\text {Normal}}(\theta _{g}^{post,s}, s_{g}^{2})$ using the draw from the posterior as the assumed mean of the normal distribution. A sample from the posterior predictive distribution of $T(\mathbf {y})$ is then computed from the individual draws $\hat{\theta} _{1}^{pred,s}, \ldots , \hat{\theta} _{G}^{pred,s}$. Figure 5 shows posterior predictive simulations for both the basic shrinkage and extended Dixon–Simon models, and for each of these models, the median, standard deviation, minimum, and maximum were chosen as the test statistics $T(\mathbf {y})$ to examine. There is no suggestion in these posterior predictive checks that either of the models is deficient in any particular way. For both models, the observed values of the test statistics appear to be quite typical values in terms of the posterior predictive distribution, though the observed standard deviation and maximum seem to be somewhat more representative of predicted values from the extended Dixon–Simon model. If the values of one or more of the test statistics $T({{\varvec{y}}})$ were nearer to the tails of the posterior predictive distribution, this would be an indication that the model should be changed in some way or that one should consider an alternative model.

## Conclusion

The Bayesian approach offers both an effective and practical framework for evaluating differences in treatment effectiveness due to heterogeneity in patient characteristics. Bayesian methods have particular advantages in the analysis of HTE as they provide a flexible framework for synthesizing evidence of all types such as prior information or information across subgroups. Despite these merits, a number of factors has limited wider adoption of Bayesian methods including lack of accessible software and concerns about the choice and impact of priors. We have addressed these concerns by outlining specific models that can be used to examine HTE and by highlighting the important issues involved in their implementation. We have also demonstrated these ideas using a case study and a software tool called **beanz**, which can be used as a web-server version or as a stand-alone R package.
